# Understanding E-Commerce Consumers’ Repeat Purchase Intention: The Role of Trust Transfer and the Moderating Effect of Neuroticism

**DOI:** 10.3389/fpsyg.2021.690039

**Published:** 2021-06-01

**Authors:** Hyeon Gyu Jeon, Cheong Kim, Jungwoo Lee, Kun Chang Lee

**Affiliations:** ^1^SKK Business School, Sungkyunkwan University, Seoul, South Korea; ^2^Economics Department, Airports Council International World, Montreal, QC, Canada; ^3^Department of Health Sciences and Technology, Samsung Advanced Institute for Health Sciences and Technology, Sungkyunkwan University, Seoul, South Korea

**Keywords:** trust transfer, neuroticism, satisfaction, repeat purchase, e-commerce

## Abstract

The dominant position of e-commerce is especially being articulated in the retailing industry once again due to several constraints that the world faces in the COVID-19 pandemic era. In this regard, this study explores the significant role of trust transfer (from offline to online) and the moderating effect of consumers’ neurotic traits in the framework of trust-satisfaction-repurchase intention in the e-commerce context based on a survey with 406 Korean e-commerce consumers. Moreover, a prediction-oriented segmentation (POS) technique combined with structural equation models (SEM) was utilized to reveal consumers’ probable hidden heterogeneous characteristics. The outcomes of the global model SEM analysis indicate that offline-online trust transference occurs in e-commerce, and the conveyed trust significantly influences satisfaction and consumers’ repeat purchase intention through satisfaction. Neuroticism also has significant positive effects on trust transfer in the global model. However, results in three subgroups generated by POS show heterogeneous characteristics that considerably differed from the global model test results. The implications from this study will be beneficial to field practitioners in the e-commerce industry in addressing the importance of trust transfer, negative neurotic traits as well as heterogeneous aspects of consumers.

## Introduction

The importance of e-commerce has been articulated for decades, along with the dramatic evolution of the internet. Moreover, e-commerce’s dominant role in the retailing industry is being re-illuminated in the COVID-19 pandemic era, which has substantially changed our everyday lives. This phenomenon brought great success to several online-based retailers (i.e., Amazon and eBay) while brought severe slump to those physical-store-based retailers (i.e., traditional department stores). Of course, most brick and mortars have been tried to transit their primary business channel into the network environment, and many of them successfully settled in the new ecosystem by conducting a multichannel strategy (i.e., Bestbuy and Walmart). In this regard, the uncertainty for these retailers would be whether consumers’ trust from online shopping experiences remain the same as they originally perceived from offline shopping experiences or not. It is evidently crucial for vendors in e-commerce to sufficiently recognize and focus on trust transfer from offline to online, especially if they have a multichannel business (Lee et al., 2007; Ke et al., 2016). Shoppers usually had experienced purchasing in offline shops where offline trust was developed. Likewise, consumers have a tendency to build online trust during the experience of frequent purchasing in e-commerce (Bock et al., 2012).

The trust-satisfaction-loyalty framework is one of the ways to explain the trust mechanism in the relationship of consumer-seller (Shapiro, 1987; Cyr, 2008; Jung et al., 2020). Based on the assumption that consumers’ trust transfers from offline to online channels, this approach argues that the greater the consumers’ trust, the greater their satisfaction with a transaction and their intention to purchase or repurchase in the vendor’s online stores (Chen and Chou, 2012; Javed and Wu, 2020). Thus, e-commerce consumers’ decisions could be supported by the loyalty framework.

Many recent studies in the IS field have suggested that consumers’ decision-making process is governed strongly by their personality traits, such as neuroticism (Mark and Ganzach, 2014; Sharif et al., 2014; Barnett et al., 2015; Dobre and Milovan-Ciuta, 2015). Neuroticism is related broadly to a general state of negative affect (Doty et al., 2013), and researchers in the psychology associated fields, such as cognitive science and neuroscience, have comprehensively scrutinized (Olvet and Hajcak, 2011; Doty et al., 2013; Aluja et al., 2015). In addition, consumers’ negative traits (i.e., neurotic) could positively or negatively influence technology use, including the employ online shopping channels (McElroy et al., 2007; Barnett et al., 2015). Nevertheless, although there are some IS studies on neuroticism’s effects (Mark and Ganzach, 2014; Sharif et al., 2014; Barnett et al., 2015; Dobre and Milovan-Ciuta, 2015), not many studies have discovered its role in the context of e-commerce settings (Yin et al., 2014).

Besides, the IS field has recently emphasized the importance of considering unobserved heterogeneity hidden in data samples to eliminate threats to the results’ validity and ensure their robustness (Becker et al., 2013; Schröder and Hruschka, 2017). Observed heterogeneity, such as gender, age, and other demographic factors, may be addressed quickly (Shao et al., 2015). In contrast, unobserved heterogeneity is difficult to assess in advance, and, therefore, threatens the results’ validity and biases empirical conclusions seriously when it remains unnoticed (Rigdon et al., 2010; Schröder and Hruschka, 2017; Wamba et al., 2017; Kim et al., 2020). In other words, there is a need to ensure the robustness of results by addressing such unobserved heterogeneity with logical and rigorous methods.

Henceforth, we were interested in investigating consumers’ unobserved heterogeneous neuroticism on the repurchase decision-making. As far as we have examined, unobserved heterogeneity has not been studied in this context until recently (see Table 1). Considering that consumers these days have various hidden characteristics, although these former studies have revealed potential factors affecting consumers’ decision-making in general based on the given domains, they might have overlooked the importance of exquisite consumer analysis. From these perspectives, we emphasized more on the hidden characteristics of consumers and tried to classified based on their unobserved heterogeneity in the framework of trust-satisfaction-repurchase intention. To achieve this, we applied a latent segmentation method on the basis of structural models which is called PLS-POS (partial least squares prediction-oriented segmentation) combining with a conventional structural equation modeling (PLS-SEM). Eventhough PLS (partial least squares) provides general results of statistical analysis, it does not show the characteristics that act as critical segments (Arenas-Gaitán et al., 2019). The PLS-POS enables measuring both the segments and parameters of belonging of the observed variables (Becker et al., 2013). By doing so, this paper fills the gap that the previous research failed in exploring the consumers’ hidden traits, which might considerably affect their decision-making.

**TABLE 1:** Previous research on trust transfer.

**Author(s)**	**Domain**	**Channel**	**NP and UH**
Liu et al., 2018	Social media communities	Online	N/A
Cao et al., 2018	Mobile payment	Online→Mobile	
Wu et al., 2016	Shopping mall	Offline	
Zhou, 2016	Mobile shopping	Online→Mobile	
Chen et al., 2015	C2C e-commerce	Online	
Chen and Shen, 2015	Social commerce	Online	
Shi and Chow, 2015	Social commerce	Online	
Yang et al., 2015	Mobile shopping	Online→Mobile	
Belanche et al., 2014	Public service	Offline→Online	
Lee et al., 2014	Cosmetics retailing	Online→Online Online→Offline Offline→Online	
Lien et al., 2014	Healthcare industry	Offline	
Wang et al., 2013	Social commerce	Online→Mobile	
Badrinarayanan et al., 2012	Cultural differences	Offline→Online	
Bock et al., 2012	Department stores	Offline→Online	
Hong and Cho, 2011	e-Retailers	Online	
Lee K.C et al., 2011	Bookstore retailing	Offline→Online	
Lee J. et al., 2011	Online shopping	Online	
Lin et al., 2011	Brokerage services	Online→Mobile	
Chen et al., 2009	C2C Online shopping	Online	
Yang et al., 2008	Online retailing	Offline→Online	
Kuan and Bock, 2007	Grocery retailing	Offline→Online	
Lee et al., 2007	Banking	Offline→Online	
Lim et al., 2006	Bookstore	Online	
Kim and Prabhakar, 2004	Banking	Online	
Pavlou and Gefen, 2004	Online auction	Online	
Stewart, 2003	Computer retailing	Online	
Doney and Cannon, 1997	Manufacturer – Retailer	Online	
This study	B2C E-commerce	Offline→Online	Yes

*NP, Negative Personality and UH, Unobserved Heterogeneity.*

The primary objective of this research is in three pillars. Firstly, finding the influence of trust transfer on consumers’ repeat purchase intention in e-commerce. Second, identifying neuroticism’s moderating effect on offline-online trust transition. Lastly, obtaining implications from the entire dataset analysis as well as segmented dataset analysis, which would produce several heterogeneity groups without a possible bias, to redefine the roles of trust transfer and neuroticism in the trust-satisfaction-repurchase framework.

## Literature Review and Hypotheses

### Consumer Trust and Transference

The proposed research question depends primarily on offline–online trust transference and, secondarily, on the consumer trust-satisfaction-loyalty framework. Therefore, first, we reviewed the literature on the consequences of trust transition on loyalty because it is, after all, related to consumers’ purchase behavior.

The objectification of trust could be a set of particular faith-related with benevolence, ability, and integrity of business partners (Kankanhalli et al., 2005; Chen and Hung, 2010; Chiu et al., 2010; Shiau and Luo, 2012), and is essential to formulate and understand consumer behavior both in online and offline commerce, as people develop trust in business transaction partners. Consumers normally possess confidence in a product from a firm when consumers endorse and trust it (Popescu and Ciurlãu, 2019; Standing et al., 2019). Many empirical researchers have emphasized trust by suggesting the trust-satisfaction-loyalty relation as a conceptual framework in the discussion of consumers’ behavior, consumers’ satisfaction, and their loyalty to the retailers in the context of purchasing goods (Fang et al., 2011; Chen and Chou, 2012; Hsu et al., 2014; Jeon and Lee, 2016a, b). The consumer loyalty framework in IS research can be briefly described as follows. Consumers with greater trust in sellers are more satisfied with the shopping process (Trivedi and Yadav, 2020). A high level of consumer contentment has a distinctively encouraging effect on their loyalty to trusted retailers and effects such behaviors as the purchase or repurchase decisions and intentions (Bhattacherjee, 2001; Liu et al., 2011). This conceptual framework represents consumers’ universal patterns of purchase decisions.

The online and offline integrated channels can increase customer satisfaction and loyalty further in either channel through the process referred to as “trust transfer” (Jeon and Lee, 2016a, b). Shifting trust is characterized as a cognitive procedure, which implies reproducing the trust from one reference to another or recognized units to unrecognized units. In particular, consumers’ perceived trust can flow between channels such as online-to-offline or offline-to-online. Stewart (2003) established a theory of trust transference in the context of retail trade, and trust transfer can be explained from two perspectives: source and process. For example, trust transfer may occur from traditional government services to e-government (Belanche et al., 2014) or from banks on the street to online banking services (Lee et al., 2007). On the other hand, if firms fail to acquire the trust from consumers, then there might be a possibility that consumers perceive notoriety, which will significantly negatively affect their purchase intention (Bratu, 2019; Jiménez-Castillo and Sánchez-Fernández, 2019). From the perspective of a process, such as communication or cognitive processes, an example of trust transfer might be a situation in which a trusted third party provides a consumer with a favorable recommendation of a bank (Lee et al., 2007). Also, this trust transfer process could be intervened by human agencies, such as marketing practitioners in the field, especially in the design stage of the online channel, which sole computer algorithms cannot replace (Klinger and Svensson, 2018; Mircicã, 2020). In this scenario, they are more likely to trust the bank’s online operation (Kim and Prabhakar, 2004). Trust transfer can be highly significant to businesses in which the outcomes eventually are linked with consumers’ loyalty through purchase intentions.

The way an organization addresses trust transference between channels is critical, particularly for e-commerce retailers who utilize omnichannel strategy with several revenue-generating channels (Lee et al., 2007), as consumers’ trust perception processes in e-commerce differ fundamentally from the process in a purely online or offline retail context. Consumers’ trust in retailers’ stores over the network also will be at a level similar to that in their offline stores because of their previous purchasing experiences (Bock et al., 2012). Thus, building consumer trust in offline channels can be an effective way for e-commerce vendors to sustain consumer loyalty. Also, firms could enhance consumer loyalty by maximizing the potential benefits and mitigating probable risks that could enhance their satisfaction (Hollowell et al., 2019). Based on this understanding of the consumer trust-satisfaction-loyalty framework and trust transference within the e-commerce context, we proposed the following five research hypotheses:

H1: Offline trust affects online trust positively from the trust transfer perspective.
H2: Offline trust affects satisfaction positively from the consumer loyalty perspective.
H3: Online trust affects satisfaction positively from the consumer loyalty perspective.
H4: Satisfaction affects repurchase intention positively from the consumer loyalty perspective.

### Neuroticism as a Source of Observed Heterogeneity

Yin et al. (2014) analyzed the influence of consumer sentiments on trust. Similarly, it is known well that online reviewers’ negative emotions are helpful in making appropriate shopping decisions (Kim and Gupta, 2012). However, these studies did not address the effects of such negative personality traits as neuroticism adequately, particularly in the transfer procedure of trust from the offline channels to the online channels in the context of e-commerce trade.

Personality traits cause each individual to demonstrate different affective responses to the same stimuli (Zabkar et al., 2017). These characteristics cause unusual behaviors that are not observable in others in the same specific situation (Bove and Mitzifiris, 2007). The personality trait of neuroticism is related strongly to adverse emotions such as apprehension and resentment (Doty et al., 2013; Barnett et al., 2015). Neuroticism can be expounded as the level of anxiety, depression, anger, embarrassment, worry, and insecurity (Barnett et al., 2015). It can be measured with the Big-five personality traits questionnaire (Norman, 1963; Digman, 1990; Goldberg, 1990) and has been studied previously (Olvet and Hajcak, 2011; Doty et al., 2013; Aluja et al., 2015). Highly neurotic users have more possibility to become vulnerable to psychopathy, tend to produce poor results in perilous tasks, and do not pursue opportunities to learn new things (Barnett et al., 2015); nonetheless, they may be more creative than the average person (Perkins et al., 2015).

Former literature has reported conflicting results about neuroticism’s effect on online shopping (McElroy et al., 2007; Svendsen et al., 2013; Sharif et al., 2014; Barnett et al., 2015; Jeon and Lee, 2016a, b). In the decision-making procedure for purchase, consumers with high neuroticism could be impulsive and buy a specific good without considering brand loyalty (Lin, 2010). On the other hand, they tend to perceive risk or uncertainty more strongly and are more sensitive to product prices during the purchase process (Zabkar et al., 2017). Given that highly neurotic consumers may be risk-averse, pessimistic, slow to adapt to change, very cautious in making purchase decisions, and likely to seek safety, neuroticism can be expected to have a more complex influence on the trust transfer procedures in e-commerce. However, the relation between neuroticism and the use of e-commerce systems has not been identified clearly (Barnett et al., 2015). Further, neuroticism has been considered rare in trust transfer studies to date (see Table 1). Therefore, to investigate its effects on consumers’ offline-online trust transfer, we proposed the following hypothesis.

H5: Neuroticism will moderate the process of transferring offline trust to online trust.

### Issue of Heterogeneity Embedded in the Data Sample

Researchers often presume completely homogeneous human behavior unconsciously (Liebana-Cabanillas and Alonso-Dos-Santos, 2017). This impetuous assumption could cause not only errors in the results, but practically unrealistic implications as well (Sarstedt et al., 2009). Errors attributable to data heterogeneity long have been an issue in empirical studies in the social and behavioral sciences (Rust and Verhoef, 2005; Becker et al., 2013) because they imperil the cogency of the results and distort implications (Becker et al., 2013).

The PLS-SEM has been considered an appreciative methodology to rigorously examine research data (Hair et al., 2017). The latent class technique (i.e., POS) helps test the theoretical framework by evaluating the structural model’s robustness that meets segmentation requirements. Moreover, this technique uses explanatory variables favorably by grouping individuals according to responses to variables in a proposed research model (Sarstedt et al., 2019).

The issue of data heterogeneity centers on the discovery of unobserved heterogeneity (Hair et al., 2016). Until recently, data heterogeneity analysis has followed conventional approaches, which are limited primarily to observed heterogeneity in the data. Researchers have attempted to discover moderating effects and divide samples into subgroups using *a priori* or contextual variables of heterogeneity observed typically, such as demographics (gender, age, and ethnicity, etc.), individual propensities (personality traits, neuroticism, etc.), and product attributes (Shao et al., 2015). The problem this approach poses is that it is valid only when the researcher can discern the observed heterogeneous variables. This issue is indispensable because dissimilarities, such as personal traits, among consumers could potentially influence their decision-making (Drugău-Constantin, 2019).

In contrast, unobserved heterogeneity can be identified only by statistical methods, not by researcher experience or prediction (Hair et al., 2016). Researchers attempt to identify unobserved heterogeneity within data by comparing the sub-groups’ differences based on the hidden attributes (Hair et al., 2016). It is suggested that erroneous conclusions might be drawn because of the absence of in-depth consideration of unobserved data heterogeneity, arguing that “There may be significant heterogeneity in the data across unobserved groups, and it can bias parameter estimates, lead to Type I and Type II errors, and result in invalid conclusions” (Becker et al., 2013). Type I errors are attributable to researchers’ ignorance of the significant differences in heterogeneous subgroups that lead to overgeneralization of the results of the entire sample. In contrast, Type II errors occur when researchers take insignificant effects without considering reversal influences in the heterogeneity groups (Becker et al., 2013). Because hidden heterogeneous traits have a substantial ability to bias results when it is not discovered, the research might obtain wrong consequences (Rigdon et al., 2010; Schröder and Hruschka, 2017; Wamba et al., 2017; Kim et al., 2020). Hence, unobserved heterogeneity should be considered when researchers perform experiments because identifying it improves the possibility that the study will yield relevant implications and offer adequate information (Becker et al., 2013). However, if unobserved heterogeneity could be detected, it can also be useful from the practical perspective, such as corporations’ marketing strategies, as it prevents biases and reveals hidden innate influences (Rigdon et al., 2010).

The argument above denotes that if researchers fail to perform the unobserved heterogeneity check, they can draw a significant number of erroneous conclusions in their studies. Nevertheless, until recently, we could find no trust transfer research that addressed this issue (see Table 1). Therefore, to ensure the robustness of our research results, we examined unobserved data heterogeneity’s presence and effects from the perspectives of trust transfer and trust-satisfaction-loyalty. For this purpose, we proposed the following hypothesis.

H6: There is hidden heterogeneity in the frameworks of trust transfer and trust-satisfaction-loyalty in the e-commerce context.

**FIGURE 1:**
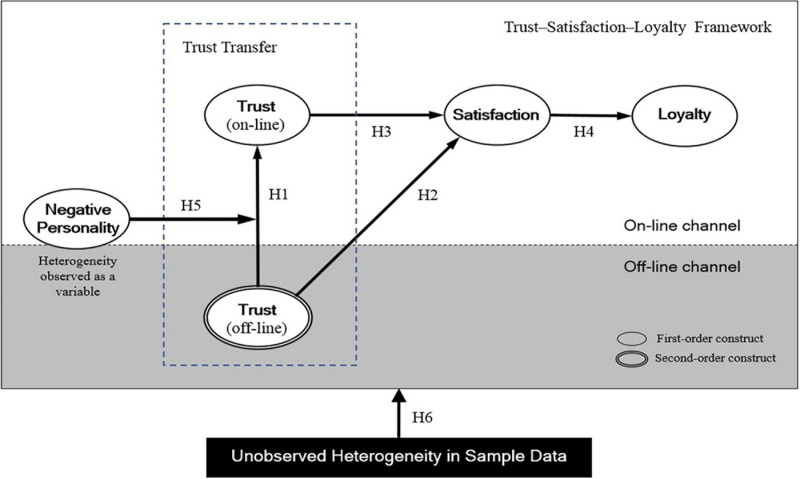
Results of PLS-SEM hypothesis testing on segmented groups according to unobserved heterogeneity (Note: TR, Trust; SA, Satisfaction; RI, Repurchase Intention; and Neuro, Neuroticism).

Figure 1 depicts the structural model containing paths representing the six hypotheses above.

## Methodology

### Sample and Data Collection

We gathered our dataset by conducting an internet-based survey with 406 consumers (females 52.5%) of Korean multichannel retailers that operate both online and offline channels. The sample data in the study were obtained by a survey company with a large panel list. Non-probability sampling was used to confirm that the participants had the previous purchasing experiences in both channels of the retailers. The participants were informed that the survey was designed to know the overall trust of consumers toward online and offline retailers.

### Measures

All measurement instruments were employed from the previous literature and validated by a pilot test with 30 samples. Five constructs were utilized, including offline trust, online trust, satisfaction, repurchase intention, and neuroticism. The questionnaire was prepared after the measurement items were corrected and modified based on the pilot results to confirm that all questions were explicit (see Table 2). To examine items, we used the scale of seven-point Likert.

**TABLE 2:** Research constructs and measurements.

**Construct**	**Item #**	**Measurement items**
Offline trust (Seppänen et al., 2007)		
*Company trust*	Comp1	The company will do what it takes to make us happy.
	Comp2	This salesperson’s company has quality people working for them.
	Comp3	The company this salesperson works for will stand behind us.
	Comp4	The company can be counted upon to do right with us.
	Comp5	The salesperson’s company has a poor reputation. (reversed)
*Product trust*	Prod1	The product will do something we want to do.
	Prod2	The product will not meet our needs without question. (reversed)
	Prod3	The product will please all those who use it or are responsible for it.
	Prod4	This product has the technical attributes necessary to do the job.
*Salesperson trust*	Sale1	The salesperson is like a good friend.
	Sale2	When the salesperson tells me something, it must be true. (reversed)
	Sale3	The salesperson did everything possible for us.
	Sale4	The salesperson is not a real expert. (reversed)
	Sale5	The salesperson will always use good judgment.
Online trust (Odekerken-Schröder et al., 2003; Teo and Yu, 2005)	TRon1	I have trust in this online store.
	TRon2	This online store gives me a trustworthy impression.
	TRon3	This online store gives me a feeling of trust.
Satisfaction (Bai et al., 2008)	SA1	I am satisfied with my decision to visit this online store.
	SA2	My choice to visit this online store was a wise one.
	SA3	I think I did the right thing by visiting this online store.
Repurchase intention (Bhattacherjee, 2001; Lee, 2010)	RI1	I will frequently use the online store in the future.
	RI2	I will use the online store regularly in the future.
	RI3	I will strongly recommend that others use it.
Neuroticism (Pervin and John, 1999; Barnett et al., 2015)	Neuro1	I am not easily bothered by things. (reversed)
	Neuro2	I rarely get irritated. (reversed)
	Neuro3	I feel comfortable with myself. (reversed)
	Neuro4	I have frequent mood swings.

“Offline trust” was adopted from Seppänen et al. (2007), using a second-order construct, covering three dimensions: firms, products, and salespeople. This multi-dimensional construct was chosen because it was more manifest in their aspects, demonstrated discriminant validity, and had reduced collinearity risk (Hair et al., 2016). Company trust is identified as the consumers’ belief toward retailers in terms of responsibilities and obligations. Product trust implies the expectations of consumers’ that the products and services of the retailers are functionally faithful. Finally, salesperson trust refers to the consumers’ beliefs that the salespeople will be faithful to their obligations.

“Online trust” is defined as that online vendors would treat consumers with high honesty. To measure the trust for the online channels, we used the questionnaire from the studies of Odekerken-Schröder et al. (2003) and Teo and Yu (2005).

“Satisfaction” implies consumers’ assessment of the extent to which vendors satisfy consumers’ needs. For the measurement, we used the questionnaire that Bai et al. (2008) have developed.

“Repurchase intention” in the online channels was assessed using the questionnaire that Bhattacherjee (2001) and Lee (2010) have developed, and “neuroticism” was evaluated using the items from the research of Barnett et al. (2015) and Pervin and John (1999).

### Procedure and Statistical Technique

The model was formulated based on the trust-satisfaction-loyalty framework, assuming that consumers’ neuroticism will affect the process of offline–to–online trust transfer (see Figure 1).

We focused first on identifying neuroticism’s effects on the relation of the transfer from offline to online trust using the entire dataset (global model). In addition, we attempted to reveal neuroticism’s unobserved effects based on three data groups segmented by the POS method that is an approach to segment data in a non-parametric procedure (Sánchez-Prieto et al., 2017).

## Results

### Measurement Model Analysis

We first examined statistical criteria for the measurement model evaluation, including internal consistency (i.e., Cronbach’s α > 0.7), composite reliability (CR > 0.7), convergent validity (i.e., average variance extracted, AVE > 0.5), collinearity diagnosis (i.e., variance inflation factor; VIF < 3.3), and discriminant validity (i.e., Fornell and Larcker criterion and heterotrait–monotrait). Tables 3–6 show that the reliability and validity of the constructs and items have been rigorously confirmed (Carmines and Zeller, 1979; Fornell and Larcker, 1981; Bagozzi and Yi, 1988; Gefen et al., 2000; Straub et al., 2004; Henseler et al., 2015; Hair et al., 2016).

**TABLE 3:** Results for measurement model.

**Scale/Items**		**Mean**	**SD**	**F/L**	**CR**	**AVE**	**α**	***R*^2^**	***t*-value**
Offline trust		0.961	0.654	0.956					
	*Company trust*	4.708	0.965		–	–	–	n/a	
	Comp1			0.884					44.418
	Comp2			0.901					54.234
	Comp3			0.898					51.375
	Comp4			0.804					31.092
	*Product trust*	4.814	0.910		–	–	–	n/a	
	Prod1			0.882					50.991
	Prod2			0.886					55.086
	Prod3			0.919					63.516
	Prod4			0.878					47.938
	*Salesperson trust*	4.889	0.913		–	–	–	n/a	
	Sale1			0.900					62.407
	Sale2			0.777					25.574
	Sale3			0.898					59.245
	Sale4			0.870					50.291
	Sale5			0.899					56.825
Online trust	4.881	1.096		0.949	0.860	0.919	0.144		
	TRon1			0.943					155.357
	TRon2			0.940					113.036
	TRon3			0.898					40.847
Satisfaction	4.537	0.853		0.933	0.822	0.892	0.336		
	SA1			0.919					90.180
	SA2			0.930					110.009
	SA3			0.871					46.514
Repurchase intention	4.349	0.977		0.909	0.769	0.849	0.485		
	RI1			0.900					67.241
	RI2			0.908					83.158
	RI3			0.820					35.525
Neuroticism	5.802	1.226		0.928	0.811	0.886	n/a		
	Neuro1			0.854					11.625
	Neuro3			0.963					17.900
	Neuro4			0.881					11.626

*Anchors for these scales are: 1 = Strongly disagree; 2 = Disagree; 3 = Slightly disagree; 4 = Neither agree nor disagree (neutral); 5 = Slightly agree; 6 = Agree; and 7 = Strongly agree.*

**TABLE 4:** Fornell and Larcker criterion for discriminant validity.

**Construct**	**1**	**2**	**3**	**4**	**5**	**6**	**7**
Offline trust	1. Company trust	0.874					
	2. Product trust	0.787	0.892				
	3. Salesman trust	0.751	0.772	0.873			
4. Online trust	0.341	0.343	0.359	0.927			
5. Neuroticism	0.099	0.119	0.132	0.151	0.9		
6. Satisfaction	0.511	0.575	0.483	0.328	0.074	0.907	
7. Repurchase intention	0.375	0.405	0.332	0.201	−0.016	0.697	0.877

**TABLE 5:** Heterotrait-monotrait ratio (HTMT) for discriminant validity.

**Construct**	**1**	**2**	**3**	**4**	**5**
1. Offline trust	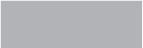				
2. Online trust	0.404	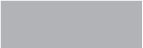			
3. Satisfaction	0.142	0.155	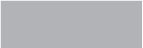		
4. Neuroticism	0.612	0.361	0.089	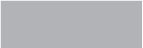	
5. Repurchase intention	0.443	0.222	0.072	0.797	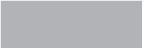

*Gray box is blank, which is the standard reporting format of PLS-SEM HTMT analysis.*

**TABLE 6:** Confidence intervals and VIF.

**Path**	**Original sample**	**Sample mean**	**2.5%**	**97.5%**	**VIF**	***R*****^2^**
Company trust → Offline trust	0.324	0.323	0.306	0.342	3.031	
Products trust → Offline trust	0.350	0.349	0.331	0.369	3.271	
Salesperson trust → Offline trust	0.410	0.409	0.390	0.427	2.858	
Offline trust → Online trust	0.353	0.352	0.240	0.460	1.023	0.180
Offline trust → Satisfaction	0.517	0.516	0.419	0.604	1.168	0.336
Online trust → Satisfaction	0.132	0.133	0.039	0.225	1.168	
Satisfaction → Repurchase intention	0.697	0.696	0.624	0.761	1.000	0.485
Neuroticism → Online trust	0.118	0.125	0.041	0.222	1.012	

### Structural Model Analysis and Hypothesis Tests

The proposed hypotheses were examined in terms of trust transfer and the consumer loyalty framework (H1 to H4) and neuroticism’s moderating effect (H5) based on the measurement model’s reliability and validity. The proposed hypotheses were supported statistically when the total number of respondents was used (*n* = 406).

In the trust transfer context, H1 test found that online trust is statistically positively influenced by offline trust (β = 0.35, *p* < 0.001). This confirms that consumers’ offline trust is moved to online. Additionally, the H2 test showed that offline trust positively and significantly affects online satisfaction (β = 0.52, *p* < 0.001). Thus, offline trust can be a substantial component that affects consumers’ decisions in e-commerce.

With respect to the consumer loyalty process in the online context, the H3 and H4 tests revealed that online trust has a significantly positive influence on online satisfaction (H3: β = 0.13, *p* < 0.01), and online satisfaction also have positive relationships with repurchase intention (H4: β = 0.70, *p* < 0.001). The results are aligned with many previous studies.

H5 is to examine whether neuroticism moderates the path between offline trust and online trust or not. To test this hypothesis, we created an interaction construct (offline trust x neuroticism) by multiplying offline trust (predictor) by neuroticism (moderator) to predict the online trust construct. The estimated coefficient of the moderating effect indicates a significance (β = 0.16, *p* < 0.05); thus, neuroticism moderates the offline-online trust relationship by increasing their degree of online trust. Table 7 below shows the results of our hypothesis tests.

**TABLE 7:** Result of hypothesis tests (*N* = 406).

**Hypothesis**	**Coefficient**	**Mean**	**SD**	***t*-values**	***P*-values**	**Results**
H1. Offline trust → Online trust	0.353	0.352	0.056	6.294	0.000	Accepted
H2. Offline trust → Satisfaction	0.517	0.516	0.047	11.010	0.000	Accepted
H3. Online trust → Satisfaction	0.132	0.133	0.048	2.754	0.006	Accepted
H4. Satisfaction → Repurchase intention	0.697	0.696	0.035	19.878	0.000	Accepted
H5. (Offline trust x Neuroticism) → Online trust	0.164	0.162	0.066	2.499	0.012	Accepted

### Revealing Unobserved Heterogeneity

Becker et al. (2013) emphasized that unobserved heterogeneity might often conceal the associations between the latent variables. Hence, the latent class methods have been called for calculating the PLS path models by recent researches (Becker et al., 2013; Mourad and Valette-Florence, 2016). In the study, to test Hypothesis 6 for uncovering unobserved heterogeneity in the data sample, we performed the PLS-POS analysis and then re-tested our hypothesis using the segmented data subgroups.

The PLS-POS method is a predictive-oriented segmentation analysis that separates data based on their unobserved heterogeneity. The technique uses a classification approach to allocate observations to clusters deterministically based on data distance (Hair et al., 2017). This part of the analysis addressed our primary purpose, to compare the effects of neuroticism (heterogeneity observed as a variable) in segmented data subgroups (discovered by unobserved heterogeneity analysis using PLS-POS). This analysis demonstrated that the initial results of the hypothesis test could not be accepted at face value.

Although PLS-POS methodology does not offer any indices to select the “best” number of segementations, the techniques relies on the assumption of a distribution-free allocation. On the contrary, the FIMIX approach assumes a multinormal distribution of latent variables, which is not likely to maintain (Mourad and Valette-Florence, 2016). Hence, we applied PLS-POS distribution-free allocation technique.

Since we should identify the number of unknown segmentations, we followed Becker et al. (2013) guidelines to apply this technique using the PLS path model (the global model) for Hypotheses 1 through 5 and calculated the solution for the number of groups (*K*). We began the POS with two segmentations (*K* = 2) and increased *K* sequentially until the proportion of one of the groups did not satisfy the prerequisite (10%). The aggregated R^2^ of the target construct was chosen in optimization settings, which corresponded to repurchase intention in our model. When the first segmenting process was complete, the original dataset was segmented into two groups (*K* = 2), the first number of predefined groups. Finally, when *K* = 7, we finished the iteration because one of the groups failed to meet the criterion with a proportion of 9.4%.

**FIGURE 2:**
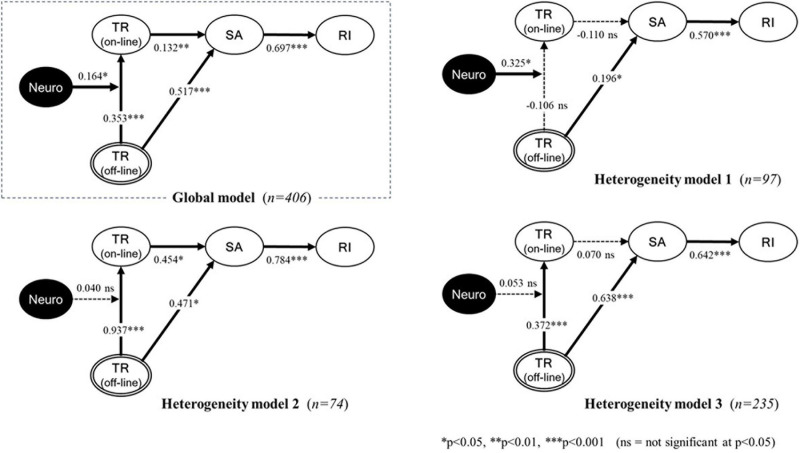
Proposed research model.

The segmentation with *K* = 3 was selected with the optimization measure (i.e., the highest average of *R*^2^). Each subgroup’s relative segment sizes of the selected segmentation (*K* = 3) were 23.9% (*n* = 97, 51.5 percent male, mean age = 40.3), 18.2% (*n* = 74, 45.9 percent male, mean age = 41.3), and 57.9% (*n* = 235, 46.4 percent male, mean age = 40.8). See Table 8 for more details. Next, we analyzed the structural models using the dataset of the generated three subgroups from POS. The hypotheses test results of each subgroup differed from the full dataset results (*n* = 406). Moreover, the PLS-POS segmented subgroups’ results were quite different (see Table 9 and Figure 2).

**TABLE 8:** PLS-POS results for segment retention criteria.

**Segment (*K*)**	**Offline trust**	**Online trust**	**Repurchase intention**	**Satisfaction**	**Σ*R*^2^**	**Sizes (relative)**
Original *R*^2^	0.999	0.144	0.485	0.336	1.964	406 (100%)
*K* = 2	0.999	0.315	0.806	0.512	1.989	290 (71.4%)
*K* = 2	0.996	0.030	0.212	0.108		116 (28.6%)
*K* = 3	0.996	0.007	0.324	0.051	2.344	97 (23.9%)
*K* = 3	0.992	0.876	0.615	0.827		74 (18.2%)
*K* = 3	0.998	0.141	0.721	0.484		235 (57.9%)
*K* = 4	0.999	0.255	0.882	0.519	2.308	226 (55.7%)
*K* = 4	0.993	0.105	0.046	0.248		63 (15.5%)
*K* = 4	0.994	0.734	0.730	0.397		69 (17%)
*K* = 4	0.995	0.415	0.841	0.081		48 (11.8%)
*K* = 5	0.998	0.530	0.457	0.263	2.067	90 (22%)
*K* = 5	0.996	0.050	0.254	0.551		73 (18%)
*K* = 5	0.990	0.053	0.648	0.443		81 (20%)
*K* = 5	0.998	0.088	0.643	0.335		81 (20%)
*K* = 5	0.996	0.184	0.597	0.261		81 (20%)
*K* = 6	0.996	0.172	0.260	0.358	1.998	68 (16.7%)
*K* = 6	0.996	0.171	0.603	0.644		67 (16.5%)
*K* = 6	0.995	0.209	0.648	0.226		68 (16.7%)
*K* = 6	0.996	0.275	0.479	0.335		69 (17.1%)
*K* = 6	0.997	0.164	0.437	0.357		66 (16.3%)
*K* = 6	0.997	0.004	0.418	0.248		68 (16.7%)
*K* = 7	0.998	0.530	0.888	0.595	2.439	65 (16.0%)
*K* = 7	0.998	0.357	0.772	0.825		48 (11.8%)
*K* = 7	0.996	0.342	0.641	0.381		59 (14.5%)
*K* = 7	0.997	0.014	0.861	0.531		38 (9.4%)
*K* = 7	0.995	0.155	0.411	0.352		48 (11.8%)
*K* = 7	0.998	0.502	0.693	0.165		81 (20.0%)
*K* = 7	0.993	0.140	0.675	0.271		67 (16.5%)

*Notes: The average *R*^2^ was highest in the seven groups segment solution (*K* = 7), but the smallest group (9.4%) did not meet the minimum sample size requirement.*

**TABLE 9:** Results of hypothesis tests for segmented groups.

**Hypothesis**	**Heterogeneity model 1 (*N* = 97)**		**Heterogeneity model 2 (*N* = 74)**		**Heterogeneity model 3 (*N* = 235)**	
	**Coeff.**	***t*-value**	**Coeff.**	***t*-value**	**Coeff.**	***t*-value**
H1. Offline trust → Online trust	−0.106	0.758	0.937	43.631***	0.372	6.155***
H2. Offline trust → Satisfaction	0.196	2.118*	0.471	2.418*	0.638	16.253***
H3. Online trust → Satisfaction	−0.110	1.121	0.454	2.362*	0.070	1.302
H4. Satisfaction → Repurchase intention	0.570	7.688***	0.784	20.085***	0.642	15.586***
H5. (Offline trust x Neuroticism) → Online trust	0.325	2.008*	0.040	0.927	0.053	0.726

*p < 0.05, ***p < 0.001.

In summary, the analysis of the entire dataset (*n* = 406) confirmed all five hypotheses. However, the analysis of the segmented dataset yielded somewhat different findings. For example, in subgroup 1 (*n* = 97), both Hypothesis 1 (Offline trust → Online trust) and Hypothesis 3 (Online trust → Satisfaction) were rejected. In subgroup 2 (*n* = 74), Hypothesis 5 (Offline trust X Neuroticism → Online trust) was rejected. In subgroup 3 (*n* = 235), both Hypothesis 3 (Online trust → Satisfaction) and Hypothesis 5 (Offline trust X neuroticism → Online trust) were rejected. These findings suggest that our first study might contain possible Type I errors (Becker et al., 2013). Table 10 shows a comparison of our research models’ key findings.

**TABLE 10:** Comparison of key findings.

**Perspectives**	**Global model**	**Model 1**	**Model 2**	**Model 3**
1. Trust transfer	O	O	X	O
2. Neuroticism effect on trust transfer	O	X	O	X
3. Effect of online trust on satisfaction after trust transfer	O	O	X	X

*Model 1 = Heterogeneity model 1, Model 2 = Heterogeneity model 2, and Model 3 = Heterogeneity model 3.*

## Discussion

The global model analysis showed that consumers who trust retailers’ offline channels also trust their online channels. The result from the global model analysis explained that consumers’ trust in offline channels transfers statistically significantly to online channels (β = 0.353, *p* < 0.001). Moreover, trust transfer influences consumers’ perceptions in the trust-satisfaction relation both in offline (β = 0.517, *p* < 0.001) and online (β = 0.132, *p* < 0.01) and satisfaction-loyalty relation positively, (β = 0.697, *p* < 0.001) and therefore, makes them willing to repurchase. These results are aligned with previous literature that explored offline-online trust transfer (Badrinarayanan et al., 2012; Bock et al., 2012; Belanche et al., 2014; Lee et al., 2014) and trust-satisfaction-loyalty (i.e., repurchase intention) relationship (Shapiro, 1987; Cyr, 2008; Chen and Chou, 2012; Javed and Wu, 2020; Jung et al., 2020).

Meanwhile, neuroticism, a negative personality trait, was related positively to consumers’ trust transfer (β = 0.164, *p* < 0.05), as Yin et al. (2014) and Zabkar et al. (2017) argued. This result indicates that there will be a higher possibility for neurotic consumers to do trust transferrence. However, in purchasing situations in general, highly neurotic consumers tend to hedge risks and uncertainties about their purchases (Zabkar et al., 2017). Hence, these consumers generally hesitate to transfer from one channel to another channel compared to consumers who are relatively less neurotic. Therefore, this study’s outcomes are fascinating and novel because of representing the unusual consequences of neuroticism on trust transfer.

Concerning our exploration of the unobserved heterogeneity that may exist within data samples, which was this study’s core research issue (RQ), the results of the global model described earlier showed the potential for biased interpretation. That is, since the global model cannot detect the characteristics that might appear in different segments, we searched the latent groups based on the global model using latent class’ techniques, in the study PLS-POS. The results showed three significant segments. It is important to check the improvement of the extracted variance (R2) when the segments are considered (Becker et al., 2013; Arenas-Gaitán et al., 2019). In Table 8, when calculating the model with the complete sample (Global), the aggregated R2 of the model is 1.964. When we consider each segment (*K*), the aggregated R2 increases significantly (Originial: 1.964; *K*2: 1.989; and *K*3: 2.344).

This implication is attributable to the following three significant differences between the model with the entire dataset and models with heterogeneous traits. Firstly, when it comes to the model with the entire dataset, it discovered the phenomenon of trust transfer, while one of the three subgroups did not. Second, the significant influence of trust on satisfaction was not found in two of the three subgroups. Finally, the effect of consumers’ neuroticism, a negative personality trait, was not significant in two of the three subgroups during the process of trust transfer.

The implications above imply that revealing consumers’ hidden characteristics is fundamental in the field of e-commerce research. Particularly, the results from the sub-models implicitly indicated that neuroticism is unrelated to, or has a weak effect on, consumers’ trust transfer, although the global model’s findings suggested that neuroticism had a significant and explicit moderating impact on consumers’ trust. Moreover, neuroticism’s influence on online channels after the consumers’ trust had transferred exhibited various patterns. Hence, in the relation between the consumers’ negative personality trait and trust-satisfaction-loyalty, the main RQ of this study, the following new implications can be derived:

The first heterogeneity model showed that consumers’ high neuroticism affects the transition of transfer significantly (β = 0.325, *p* < 0.05). On the other hand, the second and third heterogeneity models indicated clearly that consumers’ weak neuroticism does not affect trust transfer, as the trust does transfer from offline to online channels. These results demonstrate the potential to identify conflicting associations between trust transfer and neuroticism, which explains implicitly that trust in offline channels is more likely to transfer to online channels in e-commerce situations when consumers demonstrate relatively weak neuroticism. As Bove and Mitzifiris (2007) suggested, these findings show that neurotic characteristics of consumers cause unusual behaviors that might not be observed with conventional analysis.

Second, neuroticism’s effect demonstrated intriguing patterns in online channels after the trust transfer occurred. In the first heterogeneity model, the trust did not affect satisfaction significantly on the part of the consumer group that was highly neurotic, while the trust-satisfaction relation in the consumer groups that were slightly neurotic exhibited inconsistent patterns in the second (β = 0.937, *p* < 0.001) and third (β = 0.372, *p* < 0.001) heterogeneity models. These differences in the three models could be excellent supportive evidence of why researchers and field practitioners should take consumers’ hidden heterogeneities into account, as Becker et al. (2013) initially suggested. Nonetheless, in all three heterogeneity models, the “offline trust → satisfaction → repurchase intention” relation was significant, as in the global model that is consistent with former studies (Shapiro, 1987; Cyr, 2008; Chen and Chou, 2012; Javed and Wu, 2020; Jung et al., 2020). Hence, this confirmed ultimately that consumers’ loyalty in an e-commerce situation is based firmly on trust in offline channels rather than trust in online channels or consumers’ negative personality traits.

In today’s extremely competitive e-commerce business environment, it is inevitable for corporations to develop adequate strategies that are adequate for their different types of consumers. Hence, based on the outcomes, this research classifies consumers into four distinct brands in the e-commerce context: (1) carnivorous; (2) omnivorous; (3) herbivorous, and (4) frugivorous.

First, carnivorous consumers, who reflect the first heterogeneity model in this research, are a relatively sophisticated and classic type of consumer. These consumers tend to be highly neurotic, which drives them to retain their “offline trust” completely without transfer. Carnivorous consumers’ firm preference for “offline territory” primarily is analogous to the attribute of carnivorous animals that have an intense obsession with their hunting ground. For example, this type of consumer is more likely to choose not to purchase if the desired products are unavailable in offline channels. Similarly, carnivorous animals tend to choose to go hungry rather than deviate from their realm. Carnivorous consumers can easily be found in the context of luxury goods retailing because they possess a strong suspicion of replicas that may be sold in online channels.

Secondly, omnivorous consumers, who reflect the second heterogeneity model in this research, are a transitional type of consumer between carnivorous and herbivorous consumers. Omnivorous consumers’ tastes are not fastidious. Further, they do not extremely insist on “channel” to confer trust contrasted to carnivorous consumers. Therefore, omnivorous consumers would select any purchase channels with trust as long as they possess what they desire to buy. This behavioral characteristic is quite similar to the traits of omnivorous animals that are not actively selective about their diet as long as they can satisfy their hunger.

Thirdly, herbivorous consumers, who reflect the third heterogeneity model in this research, are a highly prominent type of consumers. These consumers are relatively weakly neurotic about transferring their trust to different channels and have a tendency to employ online and offline channels for different purposes. For example, they make purchase decisions by investigating the actual products in offline channels; however, they proceed with their purchases in one of several different online channels that provide the lowest price. Interestingly, trust is granted only to offline channels, even if herbivorous consumers make actual purchases in online channels. This typical trait of herbivorous consumers is equivalent to the ecological characteristic of herbivorous animals because both search for appropriate online channels and areas to satisfy their specific needs: price, design, and brand for herbivorous consumers and palatable grass for herbivorous animals.

Lastly, frugivorous consumers are a new generation who make purchases and confer trust only in online channels. Similar to carnivorous consumers, frugivorous consumers are highly neurotic, and hence, their trust remains in online channels and does not transfer. Frugivorous consumers have a characteristic similar to frugivorous animals, large primates because both are relatively the latest and most developed types on a classification basis. We have not yet identified this type of consumer in our research; however, if we consider the recent trends in retailing businesses, in which the online channel’s power overwhelming is attributable to offline channels, frugivorous consumers may appear and become a dominant type in the future.

Table 11 below summarizes our proposed consumer classification.

**TABLE 11:** Terminology proposal for consumer segmentation in the e-commerce context.

**Term**	**Channel preference**	**Trust**	**Neuroticism**	**Equivalent model**
1. Carnivorous consumers	Offline only	Offline only	Strong	Model 1
2. Omnivorous consumers	Offline and Online	Offline and Online	Weak	Model 2
3. Herbivorous consumers	Offline and Online	Offline only	Weak	Model 3
4. Frugivorous consumers	Online only	Online only	Strong	TBD

*Model 1 = Heterogeneity model 1, Model 2 = Heterogeneity model 2, and Model 3 = Heterogeneity model 3.*

In conclusion, identifying the discrepancies in the global model and the heterogeneity models using unobserved heterogeneity offers deeper reflective concern about trust transfer and negative personality traits in the consumers’ loyalty contexts.

## Conclusion

The practical implication of the study is that marketing strategies require consumers’ undisclosed heterogeneous behavior to be analyzed. In e-commerce purchasing situations, including offline, online, and mobile channels, highly complex factors, such as neuroticism, which may be manifested in unpredictable patterns and may invalidate marketers’ efforts, affect consumers’ purchase decisions and behaviors. In particular, if we consider the fact that the e-commerce business is intensely competitive today, identifying consumers’ hidden traits should not be ignored, as it may affect firms’ ability to survive. To provide solutions to unobserved heterogeneity for practitioners in the field of e-commerce, we proposed a novel approach to consumer classification with supplementary terminology: carnivorous, omnivorous, herbivorous, and frugivorous. Hence, firms and managers need to pursue a variety of methods to reveal heterogeneous patterns in their consumers’ behavior according to our proposed segmentation of consumers.

In summary, this paper contributes to academia by answering three research questions that this research proposed in the beginning: first, we revealed that there is the influence of trust transfer on consumers’ repeat purchase intention; second, neuroticism does moderate offline-online trust transfer relationship in general; third, we proved that ignoring hidden heterogeneity could be a bias by only examining the entire dataset, thus a more robust and detailed statistical analysis, such as PLS-POS approach, would be valuable to find fruitful insights in the dataset. These findings would be helpful to researchers and managers in the field of consumer studies.

Our study has the following limitations. First, it revealed unobserved heterogeneous patterns of consumers within the limited frame of trust transfer and trust-satisfaction-loyalty. Future studies need to consider the problem using more research models. Second, our study demonstrated its relevance to trust transfer by considering only a single consumer negative personality trait, neuroticism. Future studies need to find more emotional states. Finally, our study used a specific technique to identify unobserved data heterogeneity. Future research requires consideration of various other analytical methods, such as neural methods that can enhance the furtherance of consumer decision journey (Smidts et al., 2014; Mirica, 2019), to broaden our understanding of the effects of unobserved data heterogeneity.

## Data Availability Statement

The datasets presented in this article are not readily available because the datasets cannot be shared without participant’s prior consent. Requests to access the datasets should be directed to kunchanglee@gmail.com.

## Ethics Statement

The studies involving human participants were reviewed and approved by Sungkyunkwan Univetrsity IRB no. 2017-12-011-022. The patients/participants provided their written informed consent to participate in this study.

## Author Contributions

HJ designed the experiment, collected and analyzed the data, and drafted and revised the manuscript. CK assisted with the experiment, analyzed the data, and drafted and revised the manuscript. JL also assisted with data collection and its analysis. KL supervised the experimental design and the data collection and revised the manuscript. All authors contributed to the article and approved the submitted version.

## Conflict of Interest

The authors declare that the research was conducted in the absence of any commercial or financial relationships that could be construed as a potential conflict of interest.
